# Machine Learning Analysis of the Cerebrovascular Thrombi Proteome in Human Ischemic Stroke: An Exploratory Study

**DOI:** 10.3389/fneur.2020.575376

**Published:** 2020-11-05

**Authors:** Cyril Dargazanli, Emma Zub, Jeremy Deverdun, Mathilde Decourcelle, Frédéric de Bock, Julien Labreuche, Pierre-Henri Lefèvre, Grégory Gascou, Imad Derraz, Carlos Riquelme Bareiro, Federico Cagnazzo, Alain Bonafé, Philippe Marin, Vincent Costalat, Nicola Marchi

**Affiliations:** ^1^Institut de Génomique Fonctionnelle, Univ. Montpellier, UMR 5203 CNRS - U 1191 INSERM, Montpellier, France; ^2^Diagnostic and Interventional Neuroradiology Department, Gui de Chauliac Hospital, Montpellier, France; ^3^I2FH, Institut d'Imagerie Fonctionnelle Humaine, Gui de Chauliac Hospital, Montpellier, France; ^4^BioCampus Montpellier, CNRS, INSERM, Université de Montpellier, Montpellier, France; ^5^Santé Publique: Epidémiologie et Qualité des Soins, CHU Lille, University of Lille, Lille, France

**Keywords:** stroke, thrombus, cerebrovascular, mechanical thrombectomy, proteome, support vector machine learning, neuroradiology

## Abstract

**Objective:** Mechanical retrieval of thrombotic material from acute ischemic stroke patients provides a unique entry point for translational research investigations. Here, we resolved the proteomes of cardioembolic and atherothrombotic cerebrovascular human thrombi and applied an artificial intelligence routine to examine protein signatures between the two selected groups.

**Methods:** We specifically used *n* = 32 cardioembolic and *n* = 28 atherothrombotic diagnosed thrombi from patients suffering from acute stroke and treated by mechanical thrombectomy. Thrombi proteins were successfully separated by gel-electrophoresis. For each thrombi, peptide samples were analyzed by nano-flow liquid chromatography coupled to tandem mass spectrometry (nano-LC-MS/MS) to obtain specific proteomes. Relative protein quantification was performed using a label-free LFQ algorithm and all dataset were analyzed using a support-vector-machine (SVM) learning method. Data are available via ProteomeXchange with identifier PXD020398. Clinical data were also analyzed using SVM, alone or in combination with the proteomes.

**Results:** A total of 2,455 proteins were identified by nano-LC-MS/MS in the samples analyzed, with 438 proteins constantly detected in all samples. SVM analysis of LFQ proteomic data delivered combinations of three proteins achieving a maximum of 88.3% for correct classification of the cardioembolic and atherothrombotic samples in our cohort. The coagulation factor XIII appeared in all of the SVM protein trios, associating with cardioembolic thrombi. A combined SVM analysis of the LFQ proteome and clinical data did not deliver a better discriminatory score as compared to the proteome only.

**Conclusion:** Our results advance the portrayal of the human cerebrovascular thrombi proteome. The exploratory SVM analysis outlined sets of proteins for a proof-of-principle characterization of our cohort cardioembolic and atherothrombotic samples. The integrated analysis proposed herein could be further developed and retested on a larger patients population to better understand stroke origin and the associated cerebrovascular pathophysiology.

## Introduction

Stroke is a major public health burden and the second most common cause of death worldwide (1–3). Currently, the incomplete molecular understanding of stroke pathophysiology negatively impacts patients' management, follow-up, and secondary prevention (3, 4). A recent consensus indicates that examinations of patients' intracranial thrombi could help unveil novel disease mechanisms (5). Studying the intracranial thrombi composition could advance our knowledge of the molecular mechanisms of local cerebrovascular cell damage in this disease setting (6–9).

Mechanical thrombectomy (MT) is a standard of care for patients presenting with acute ischemic stroke (AIS) due to large vessel occlusion (LVO) (10). MT allows the retrieval of cerebral thrombi from brain arteries, enabling subsequent samples storage and analysis. A few studies have analyzed the histological composition of intracranial thrombi (11, 12), describing architecture or reporting the presence of fibrin and leucocytes (13). However, an in depth characterization of the thrombi molecular components is currently lacking (11).

Here, we performed a quantitative proteomic analysis of intracranial thrombi retrieved using MT from a cohort of *n* = 32 cardioembolic and *n* = 28 atherothrombotic diagnosed AIS patients. We resolved the thrombi proteomes for our cohort samples and next applied a support-vector machine (SVM) learning approach to estimate whether specific sets of proteins, alone or in combination with available clinical data, could help differentiate the cardioembolic from atherothrombotic origin in our selected population.

## Materials and Methods

### Inclusion Criteria

Patients with suspected ischemic stroke secondary to an LVO were prospectively recruited at a high-volume, comprehensive stroke center in France. Patients were required to present imaging evidence of occlusion of the internal carotid artery (ICA, cervical or intracranial part), the M1 or M2 branches of the middle cerebral artery (MCA), the basilar artery, or a tandem atheromatous occlusion defined by the occlusion of both cervical carotid artery and intracranial artery (carotid artery or MCA). Use of intravenous thrombolysis (IVT) treatment was allowed and administrated according to current guidelines (10). Stroke cause was defined by a stroke neurologist blinded to the proteomics analysis, according to the TOAST (Trial of ORG 10172 in Acute Stroke Treatment) (14) classification, after an exhaustive in-hospital workup (15) including at least computed tomography and magnetic resonance imaging, duplex sonography of the cervical arteries, blood coagulation tests, long-term electrocardiography, and transthoracic or transesophageal echocardiography. Stroke etiology was defined as “atherothrombotic tandem” when CT angiography and MR angiography demonstrated >50% stenosis or occlusion of the cervical carotid artery with associated intracranial ICA or MCA occlusion ipsilateral to the symptomatic hemisphere, in addition to exclusion of potential sources of cardiac embolism. Stroke etiology was defined as “cardioembolic” when at least one cardiac source for an embolus was identified after a complete cardiological work-up including Holter monitoring and echocardiography, in the absence of any stenosis of ipsilateral large extracranial arteries or atherosclerosis, excluding atrial fibrillation with non-cardioembolic strokes.

Exclusion criteria for the present study were: (1) failure of thrombus retrieval (failure of catheterization, patients with spontaneous reperfusion at the beginning of the procedure), (2) patients non-suitable for MT with a pre-stroke modified Rankin Scale (mRS) score of >3; (3) patients with non-atheromatous or non-cardioembolic tandem occlusions (intimal dysplasia/web, dissection), (4) patients having had MT but with a thromboembolic material unsuitable for proteomic analyses (mainly due to insufficient material amounts retrieved), (5) patients with no clear etiology or “undefined etiology” (defined as at least two possible etiologies found after a complete clinical, laboratory, and imaging work-up).

The study was approved by the local ethics committee, with the patients providing written informed consent in acute phase whenever possible. Otherwise, the consent form was signed by the patient's relatives.

### Patient Characteristics

Patient demographics, vascular risk factors, imaging data, vital signs before treatment, severity of ischemic stroke, and clinical outcomes were collected with a structured questionnaire. Age, sex, cardiovascular risk factors (hypertension, dyslipidemia, diabetes mellitus, and smoking habits), time of symptom onset, National Institutes of Health Stroke Scale (NIHSS) at baseline, use of IVT, and its time from symptom onset were collected (see Table 1). The Alberta Stroke Program Early CT Score (ASPECT) on diffusion-weighted magnetic resonance or CT imaging was assessed by a neuroradiologist.

**Table 1:** Patients data, Treatment Characteristics, Complications, and Outcomes according to stroke etiology.

**Characteristics**	**Cardioembolic (*n =* 32)**	**Atherothrombotic (*n =* 28)**	***P*-Values**
Age, years	79.5 (72.0–85.5)	67.5 (57.5–77.5)	0.005
Men	18/32 (56.3)	20/28 (71.4)	0.22
Medical history			
Hypertension	16/32 (50.0)	20/28 (71.4)	0.091
Diabetes	6/32 (18.8)	3/28 (10.7)	0.48
Hypercholesterolemia	10/32 (31.3)	8/28 (28.6)	0.82
Current smoking	6/32 (18.8)	10/28 (35.7)	0.14
Alcohol abuse	3/32 (9.4)	4/28 (14.3)	0.70
Coronary artery disease/Myocardial Infarction	5/32 (15.6)	3/28 (10.7)	0.71
Previous stroke or TIA	8/32 (25.0)	2/28 (7.1)	0.088
Cardiac failure	6/32 (18.8)	0/28 (0.0)	0.047
Coronary stent	1/32 (3.1)	1/28 (3.6)	NA
Coronary bypass	1/32 (3.1)	1/28 (3.6)	NA
Carotid endarterectomy	0/32 (0.0)	0/28 (0.0)	NA
Atrial fibrillation	20/32 (62.5)	1/28 (3.6)	<0.001
Previous antithrombotic medications	20/32 (62.5)	11/28 (39.3)	0.073
Aspirin	10/32 (31.3)	8/28 (28.6)	0.82
Clopidogrel	1/32 (3.1)	2/28 (14.3)	NA
Vitamin K Antagonist	7/32 (21.9)	0/28 (0.0)	0.0110
New oral anticoagulant	3/32 (9.4)	0/28 (0.0)	NA
Current stroke event			
Systolic blood pressure, mmHg	136 (111–151)	152 (139–170)	0.006
Diastolic blood pressure, mmHg	80 (65–90)	90 (76–96)	0.033
Heart rate	76 (64–90)	80 (70–96)	0.51
Weight, kg	71 (60–81)	79 (64–88)	0.18
Body Mass Index	24.4 (23.0–27.7)	26.0 (23.5–30.2)	0.23
Glycemia, mmol/L	6.8 (6.2–7.5)	6.8 (5.7–8.1)	0.77
NIHSS score	19 (10–24)	19 (14–23)	0.85
Pre-stroke mRS≥1	6/32 (18.8)	2/28 (7.1)	0.26
ASPECTS	8 (6–10)	8 (7–9)	0.84
Site of occlusion			
MCA (M1 or M2)	18/32 (56.3)	2/28 (7.1)	<0.001
Intracranial ICA	11/32 (34.4)	1/28 (3.6)	
Tandem	1/32 (3.1)	24/28 (85.7)	
Basilar artery	1/32 (3.1)	0/28 (0.0)	
Cervical ICA	1/32 (3.1)	1/28 (3.6)	
Complete blood count			
Hemoglobin	13.5 (12.4–14.9)	13.8 (12.8–14.7)	0.65
Platelets	203 (177–237)	250 (212–301)	0.011
White cells	8.9 (6.6–12.0)	10.3 (8.5–11.6)	0.15
Treatment characteristics			
Intravenous rt-PA	13/32 (40.6)	15/28 (53.6)	0.32
General anesthesia	9/32 (28.1)	15/28 (53.6)	0.45
Onset to groin puncture time, min	173 (147–327)	289 (184–693)	0.034
Onset to Intravenous rt-PA, min	130 (110–180)	130 (105–180)	0.58
Total number of passes	1 (1–2)	1 (1–2)	0.55
Reperfusion success	29/32 (90.6)	28/28 (100.0)	NA
Groin puncture to reperfusion, min	40 (31–59)	72 (50–97)	0.002
Adverse events			
Per-procedural complication	1/20 (5.0)	3/20 (15.0)	NA
Any ICH	12/32 (37.5)	11/28 (39.3)	0.89
PH or symptomatic ICH	0/32 (0.0)	0/32 (0.0)	NA
Functional outcome			
Favorable outcome (mRS 0–2)	10/32 (31)	14/28 (50)	0.14

*Values expressed as no/total no. (%), or median (interquartile range)*.

*ASPECTS, Alberta stroke program early computed tomography score; CT, computed tomography; ICA, internal carotid artery; ICH, Intracranial hemorrhage; MCA, middle cerebral artery; mRS, modified Rankin scale; NIHSS, national institutes of health stroke scale; PH, parenchyma hematoma; rt-PA, recombinant tissue plasminogen activator; TIA, transient ischemic attack*.

### Endovascular Procedure

All patients were treated in a dedicated neuroangiography suite under general anesthesia or conscious sedation, after evaluation by the anesthesiology team.

Most of the procedures were performed using the Trevo® device (Stryker, Kalamazoo, Michigan) or the Solitaire FR™ device (Medtronic, Dublin, Ireland) via the femoral artery approach. A balloon catheter was positioned in the ICA to allow flow arrest during thrombus retrieval. The stent retriever was delivered through a microcatheter and deployed across the thrombus. A distal aspiration during the stent retrieval was performed, according to the SAVE technique (16). A control angiogram was obtained to assess recanalization and reperfusion. This sequence was repeated until mTICI 2b or mTICI 2C/3 flow (defined as successful reperfusion) was established (17). The “retrograde approach” (also known as the distal-to-proximal or intracranial-first approach), aiming to recanalize the distal and symptomatic intracranial occlusion before addressing the cervical carotid lesion, was generally chosen for tandem occlusions. The interventional neuroradiologist used another thrombectomy device in the case of reperfusion failure (mTICI <2b) with the first stent retriever. Reperfusion results were reported by using the mTICI score (18). Peri-procedural complications [embolization in a new territory (defined as an angiographic occlusion in a previously unaffected vascular territory observed on the angiogram after clot removal), arterial dissection or perforation, vasospasm, and subarachnoid hemorrhage] were recorded.

### Follow-Up and Outcome

All patients underwent cross-sectional imaging (computed tomography or magnetic resonance imaging) within a range of 18–24 h after the procedure. Intracranial hemorrhage was classified according to the ECASS (European Cooperative Acute Stroke Study) criteria (19). Symptomatic intracranial hemorrhage was defined as any intracerebral hemorrhage with an increase of at least four NIHSS points within 24 h, or resulting in death. The mRS at 90 days was assessed by trained research nurses unaware of the study group assignments, during face-to-face interviews, or via telephone conversations with the patients, their relatives, or their general practitioners.

### Collection and Processing of Intracranial Thrombi

**Figure 1:**
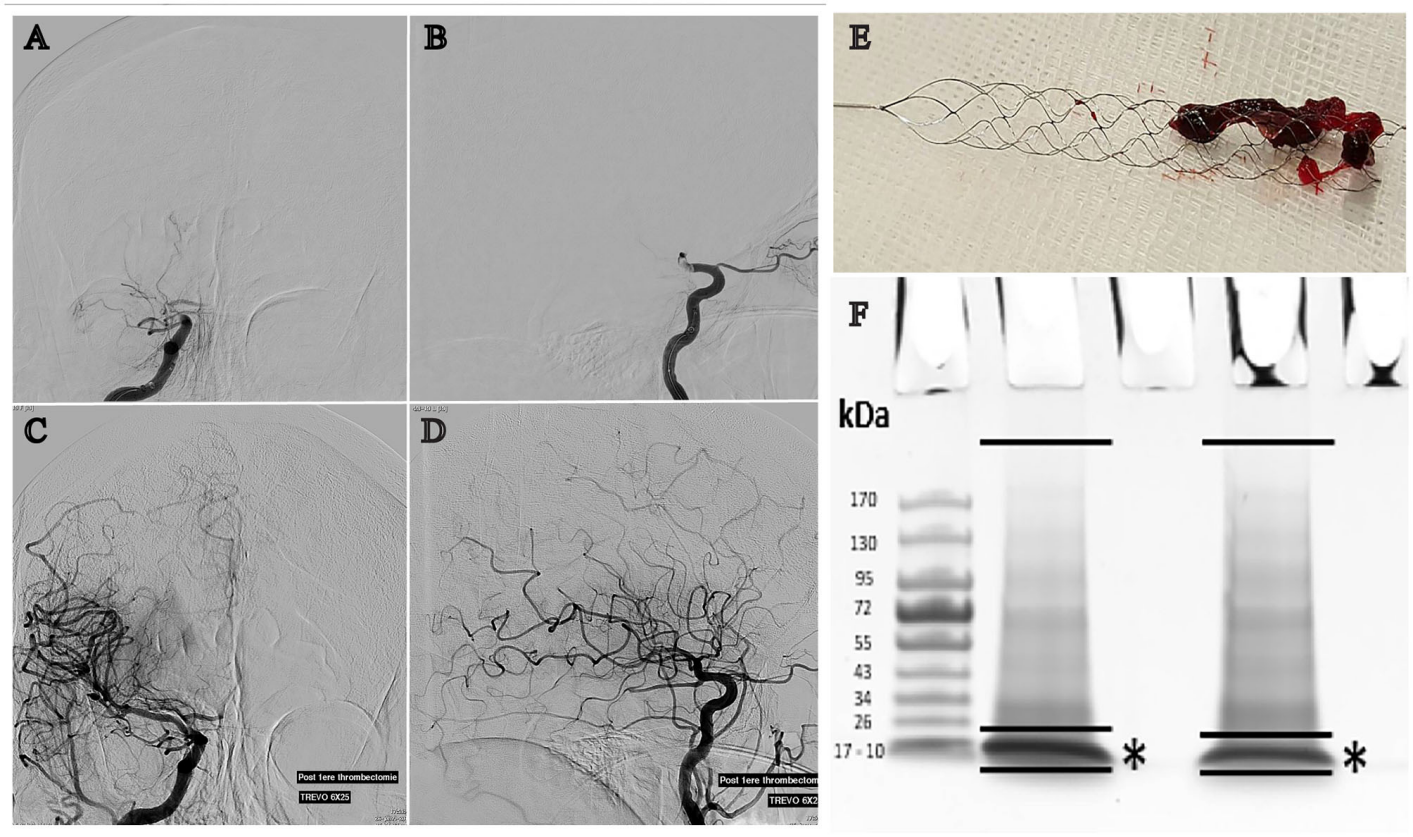
Cerebral angiography showing a right MCA occlusion before (**A**: anteroposterior projection, **B**: lateral projection) and after recanalization (**C**: anteroposterior projection, **D**: lateral projection) achieved by mechanical thrombectomy. **(E)** Clot engaged by the Trevo® stent-retriever. **(F)** Illustration of thrombi protein separation on 4–15% polyacrylamide gels and Hemoglobin depletion (*) prior to in-gel protein digestion by trypsin.

In the angiography room, after retrieval (Figure 1E), thrombi were immediately frozen at −80°C in a dedicated transportable azote freezer (Voyager, Air Liquide). In the laboratory, samples were prepared for mass spectrometry analysis. After initial mashing in a glass potter at 4°C in RIPA buffer, thrombi were further dissolved using an ultrasonic liquid processor (10 applications of 1 second each at 4°C; Vibra-cell VCX130PB, VWR) and then centrifuged (Eppendorf 5427R) at 1,200 RPM for 10 min at 4°C. Protein concentration was assessed by a bicinchoninic acid (BCA) assay. Protein extracts (20 μg) were separated by SDS-PAGE using a short (2 cm) migration. Single pieces of gel including separated proteins except hemoglobin were excised for each sample and proteins were digested in-gel using Trypsin (Trypsin Gold, Promega), as previous described (20).

### Mass Spectrometry

The resulting peptide samples were analyzed online using Q-Exactive HF mass spectrometer coupled with an Ultimate 3000 RSLC (Thermo Fisher Scientific) fitted with a stainless-steel emitter (Thermo Fisher Scientific). Desalting and pre-concentration of samples were performed online on a Pepmap® pre-column (0.3 mm × 10 mm, Thermo Fisher Scientific). A gradient consisting of 2–40% of buffer B in 123 min, then 90% of buffer B during 5 min (A: 0.1% formic acid in water; B: 0.1% formic acid 80% ACN) at 300 nL/min was used to elute peptides from the capillary reverse-phase column (0.075 mm × 500 mm, Pepmap® C18, Thermo Fisher Scientific). Spectra were acquired with Xcalibur software (v4.1 Thermo Fisher Scientific). MS/MS analyses were performed in a data-dependent mode with standard settings. MS data analysis was performed using the MaxQuant software with default settings (v1.5.5.1) (21). All MS/MS spectra were searched using the Andromeda search engine (22) against the UniProtKB Reference proteome UP000005640 database for Homo sapiens (release 2019_03, https://www.uniprot.org/) and the contaminant database in MaxQuant. Default search parameters were applied. Oxidation (Met) and Acetylation (N-term) were used as variable modifications and Carbamidomethyl (Cys) was used as fixed modification. FDR was set to 1% for peptides and proteins. A representative protein ID in each protein group was automatically selected using an in-house bioinformatics tool (Leading v3.2). First, proteins with the most numerous identified peptides were isolated in a “match group” (proteins from the “Protein IDs” column with the maximum number of “peptides counts”). For the match groups where more than one protein ID were present after filtering, the best annotated protein in UniProtKB [reviewed entries rather than automatic ones, highest evidence for protein existence, most annotated protein according to the number of Gene Ontology Annotations (UniProtKB-GOA, made on 20190416)] was defined as the “leading” protein. Graphical representation and statistical analysis of MS/MS data were performed using Perseus (v1.6.1.1). Label-free quantification (MaxQuant LFQ) was used to highlight proteins differentially expressed between samples (23).

The mass spectrometry proteomics data have been uploaded to the ProteomeXchange Consortium via the PRIDE partner repository with the dataset identifier PXD020398 (24).

### Data Analysis

Descriptive Analysis

Data in Table 1 are presented as median (range) for quantitative variables, and percentage (count) for categorical variables. Baseline and treatment characteristics, complications and outcomes were compared according to stroke etiology using Chi-Square or Fisher's exact tests for categorical variables and the Mann-Whitney *U-*test for quantitative variables. No statistical comparisons were done for categorical variables with frequency <5. Statistical testing was done at the 2-tailed α level of 0.05. Data were analyzed using the SAS package, release 9.4 (SAS Institute, Cary, NC).

A support-vector machine (SVM) approach was implemented using MATLAB (r2018a, MathWorks, Natick, MA, USA). The SVM algorithm analyzes and learns from the dataset (Supplementary Table 2) to identify the hyperplanes for the best segregation of data according to a known discriminatory characteristic (25). In our work, the relatively small sample size prevents from achieving a correct validation step and SVM was used as a statistical tool to examine whether hyperplanes exist splitting our two groups. Here, we specifically tested whether samples segregation is attainable using combinations of up to 3 proteins (trios) from those commonly detected in all samples. Each possible combinations of three proteins from the data set in Supplementary Table 2 was tested (*n* = 13,908,836), the corresponding X/Y/Z hyperplanes were defined by the SVM (see **Figure 3A**), and the percentage of correct sample classification was obtained. The protein combinations achieving the best discriminatory score for our populations were retained. SVM analysis was also performed using clinical data in Table 1.

## Results

### Clinical Data, Peripheral Blood and Thrombi Characteristics

Baseline clinical data, treatment characteristics, early complications and outcomes are provided in Table 1. In the selected population, subjects suffering from atherothrombotic stroke were younger (67.5 vs. 79.5 years old, *p* = 0.005), presented no cardiac failure (0 vs. 18.8%, *p* = 0.047), no significant atrial fibrillation (3.6 vs. 62.5%, *p* < 0.001), and displayed higher systolic and diastolic blood pressure at admission (152 and 90 mmHg vs. 136 and 80 mmHg, *p* = 0.006 and 0.033). M1 occlusions were more frequent in the cardioembolic group (56.3 vs. 7.1%, *p* < 0.001). Groin puncture to reperfusion time was longer in the atherothrombotic group, which included 85.7% of tandem occlusions (72 vs. 40 min., *p* = 0.002). Complete blood count at admission indicated that platelet levels were higher in the atherothrombotic group (250 × 10^9^/L vs. 203 × 10^9^/L, *p* = 0.011; Table 1). Weight of the retrieved thrombi was 31.2 mg for the cardioembolic group (range 5.8–206.2 mg) and 36 mg for the atherothrombotic group (range 3.2–136.2; *p* = 0.85). Total protein concentrations were 11.20 μg/μl (5.3–22.1) and 11.1 μg/μl (4–26.5; *p* = 0.82) for the cardioembolic and atherothrombotic groups, respectively.

### Analysis of the Intracranial Human Thrombi Proteome

**Figure 2:**
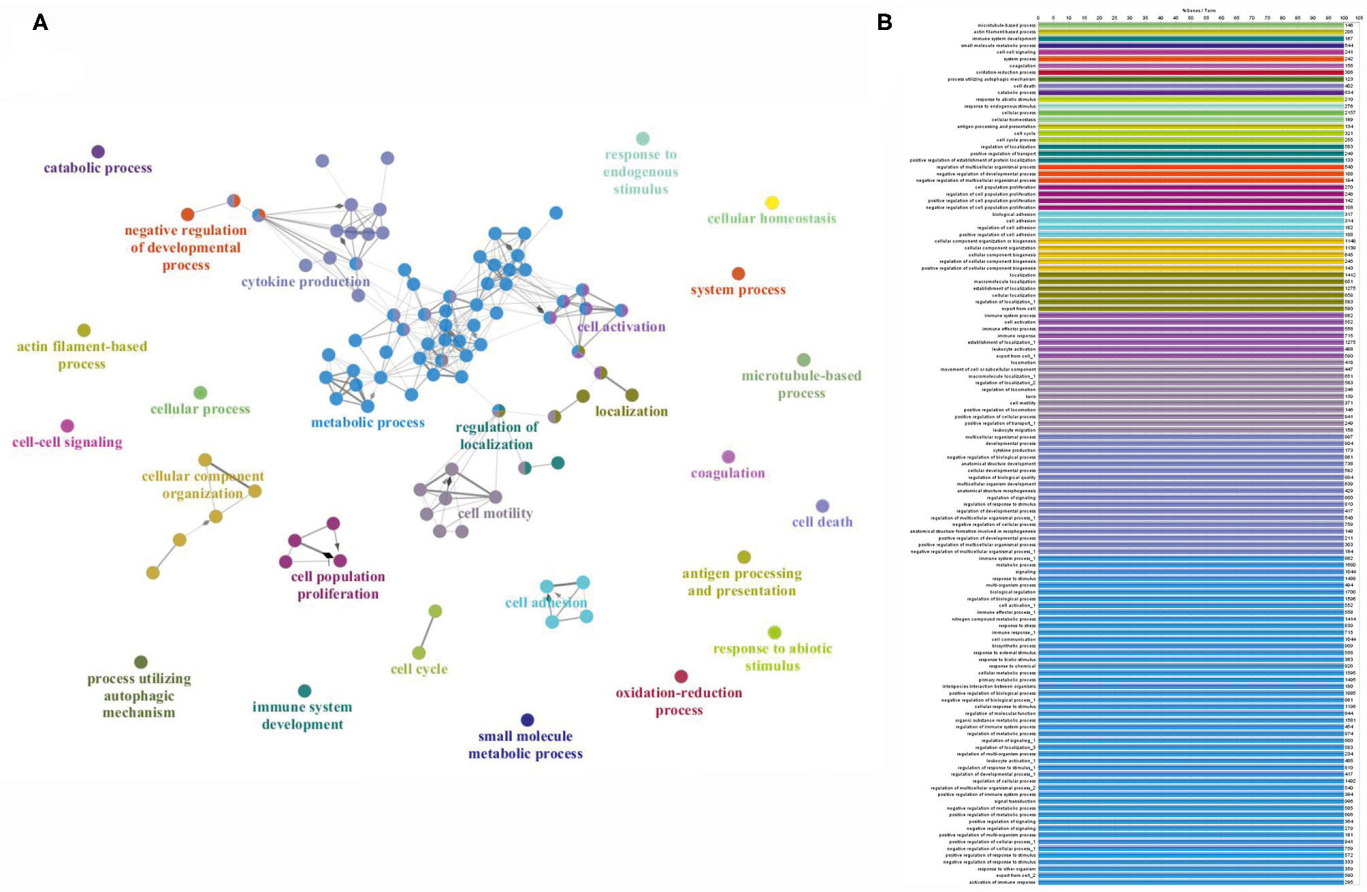
**(A)** Graphic representation of the proteome fingerprint and protein clustering according to cellular functions. **(B)** List of specific cellular processes color-coded to panel A (high resolution zoom-in).

All thrombus samples were individually processed by SDS-page chromatography and the hemoglobin band excised (Figures 1 A–F). Mass spectrometry analysis identified a total of 2,455 proteins in the samples analyzed. The complete list of all proteins detected in each sample is provided in Supplementary Table 1. A total of 438 proteins were commonly present in all the samples analyzed (Supplementary Table 2). Analysis of ClueGO annotations of the thrombi proteome, according to UniProtKB or EBI GOA databases, showed protein clusters for key biological pathways including metabolic processes, cytokines production, and cell proliferation, activation, or motility (Figure 2A). Indicating an inflammatory track are proteins associated with leukocytes activation, migration, and cell adhesion (Figure 2B; *high definition zoom-in*). This dataset constitutes the largest human thrombus proteome available and a shared library for the investigation of the molecular mechanisms of thrombus formation and ischemic stroke pathophysiology.

### Exploring the Use of Support-Vector-Machine Learning to Analyse the Thrombi Proteome

**Figure 3:**
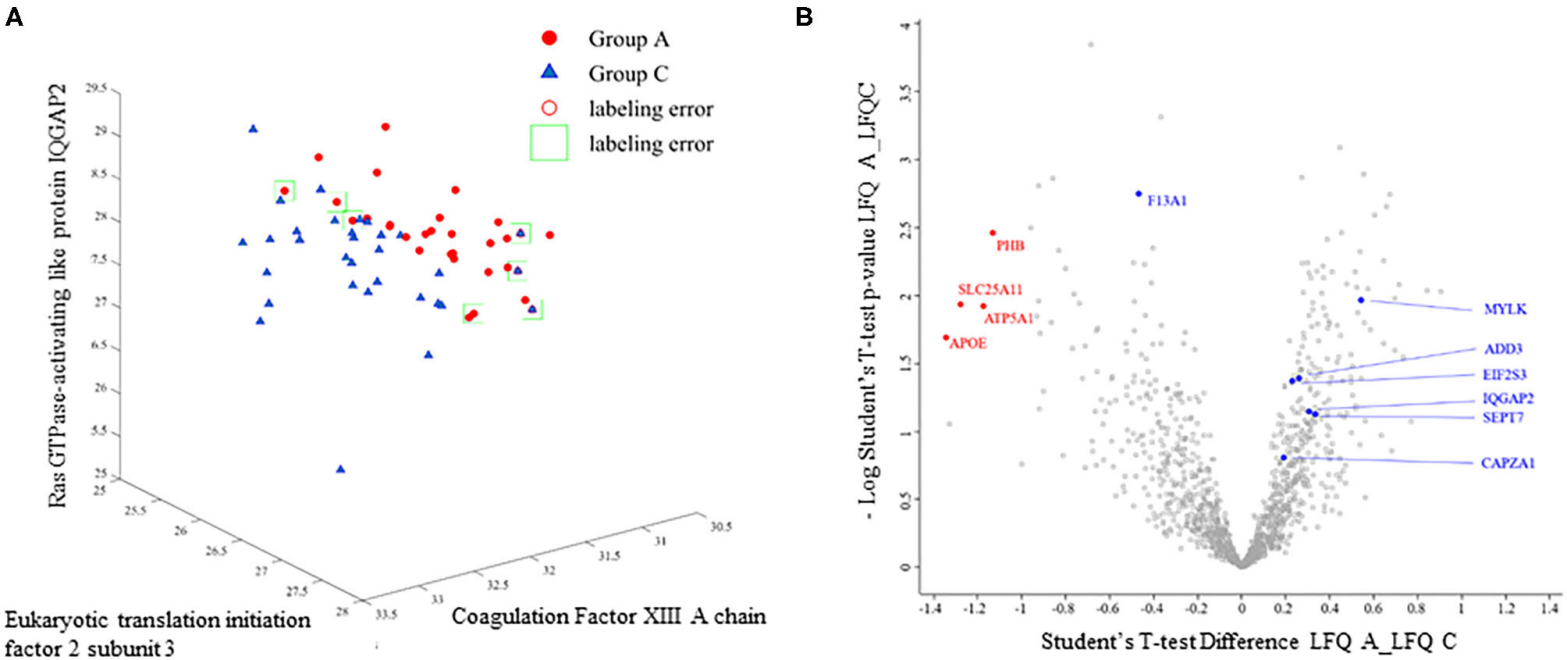
**(A)** Example of SVM plot and classification for the combination of Eukaryotic translation initiation factor 2 subunit 3, Ras GTPase-activating-like protein IQGAP2, and coagulation Factor XIII. Groups A: atherothrombotic; Group C: cardioembolic. Light green squares and red circles indicate labeling errors for misclassified patients. **(B)** Volcano plot showing proteins significantly (*p* < 0.05) enriched using LFQ values log2 transformed (red dots; see Table 3 for protein details). Student's *t-*test is performed by using Perseus algorithms. Blue dots indicate the SVM-identified proteins (see Table 2 for details).

The proteomic LFQ data obtained from our samples cohort were analyzed using a SVM routine to mathematically examine potential signatures existing between the cardioembolic and atherothrombotic proteomes. The SVM algorithm does not handle missing data across samples and the analysis was performed using the proteins commonly detected in all thrombi (438 proteins; Supplementary Table 2). In our SVM study we specifically aimed at identifying small set of discriminatory elements, here up to 3 proteins (see Methods). As a result, proteins trios were found by SVM providing a 88.3% accuracy of correct classification of our two sample groups. Proteins and their biological functions are detailed in Table 2. Factor XIII, which catalyzes the last step of the coagulation cascade by crosslinking fibrin fibers, was present in all combinations. Figure 3A shows an illustration of the SVM hyperplane classification for the cardioembolic and atherothrombotic samples according to the protein trio Eukaryotic translation initiation factor 2 subunit 3, Ras GTPase-activating-like protein IQGAP2, and Coagulation factor XIII. Using this specific setting, four and three patients were misclassified (light green squares in Figure 3A) as cardioembolic and atherothrombotic, respectively. In univariate analysis (Wilcoxon test), the coagulation Factor XIII, the Eukaryotic translation initiation factor 2 subunit 3, and the Myosin light chain kinase levels were significantly different between the cardioembolic and atherothrombotic groups, with respective *p-*values of 0.002, 0.04, and 0.01 (see Table 2). These results have a dual value, suggesting potential molecular differences between cardioembolic and atherothrombotic thrombi while supporting the notion of protein biomarkers to understand clot origin.

**Table 2:** SVM protein trios allowing 88.3% accuracy of correct classification of cardioembolic and atherothrombotic thrombi.

**Trio combinations**	**Proteins**	**Protein function (40)**	**Univariate *p-*value (bilateral Wilcoxon rank sum test)**
1	• Coagulation factor XIII	Catalyzes the last step of the coagulation cascade by crosslinking fibrin fibers	0.0022
	• Eukaryotic translation initiation factor 2 subunit 3	Involved in the early steps of protein synthesis	0.0439
	• Ras GTPase-activating-like protein IQGAP2	Binds to activated CDC42 and RAC1. Associates with calmodulin	0.1326
2	• Coagulation factor XIII	Catalyzes the last step of the coagulation cascade by crosslinking fibrin fibers	0.0022
	• F-actin-capping protein	Binds to the fast-growing ends of actin filaments in a Ca^2+^ independent manner	0.1752
	• Myosin light chain kinase	Calcium/calmodulin-dependent kinase implicated in smooth muscle contraction via phosphorylation of myosin light chains	0.0142
3	• Coagulation factor XIII	Catalyzes the last step of the coagulation cascade by crosslinking fibrin fibers	0.0022
	• Septin-7	Filament-forming cytoskeletal GTPase. Required for normal organization of the actin cytoskeleton	0.1897
	• Gamma-adducin	Membrane-cytoskeleton-associated protein that promotes the assembly of the spectrin-actin network.	0.0589

### Integrating SVM Analyses of Clinical Data and Thrombi Proteome

In an attempt to identify additional SVM differentiation factors, we performed an analysis using patients clinical data (Table 1; age, sex, history of cardiac failure or atrial fibrillation, previous antithrombotic medication, glycemia, weight and BMI, thrombus weight and global protein concentration, hemoglobin, leucocytes, and platelet rate). SVM identified history of cardiac failure and atrial fibrillation as variables differentiating the two population with an 81.36% accuracy. This result is obvious considering our study design and because history of cardiac failure was used as one of the criteria to diagnose etiology at enrollement (see Methods). Cardiac failure and atrial fibrillation are two known risk factors linked to cardioembolic stroke (3). Interestingly, when atrial fibrillation was excluded from the SVM analysis, patient age and thrombus protein concentration provided a differentiation level of 74.58% within our sample cohorts. The latter results indicate thrombus total protein concentration as a new SVM analytical variable. Addition of a third variable did not improve the SVM score (*not shown*). We do acknowledge that combining the protein trio 1 (see Table 2), history of cardiac failure, and protein concentration we obtained a SVM score of 96.6%.

### Testing Proteome Using LFQ Statistics

The selected SVM method tests all combinations of three inter-dependent proteins, obtaining solutions for data clusterization that are not executable using LFQ and standard statistics (26). Thus, a Student's *T-*test (Perseus algorithms) analysis on the proteins (log2 transformed) detected in all samples did not deliver significant difference between the studied cardioembolic and atherothrombotic populations. Furthermore, we applied a conventional method where proteomes (Supplementary Table 1) are filtered to include proteins with at least 50% of valid LFQ values. By using this approach, Student's *t-*test identified four proteins (PHB, SLC25A11, ATP5A1, and APOE; see Table 3) that display an abundance in cardioembolic as compared to atherothrombotic thrombi (volcano plot in Figure 3B). However, LFQ *T-*test difference was low (x-axis = −1.2; red dots in Figure 3B) with the crucial caveat that, because of method design, these proteins were undetectable in an elevated number of samples (Supplementary Table 1), therefore impeding group discrimination. These results support the relevance and the efficiency of SVM to analyze the proteome thombi dataset in our experimental settings.

**Table 3:** LFQ (log2 transformed) of single proteins enriched in cardioembolic as compared to atherothrombotic thrombi.

**Gene**	**Uniprot Identification (40)**	**Protein name**	**Main known protein function (40)**	**Subcellular Location**	**Student's *T*-test Difference Log2 LFQ intensity Atheroma_Log2 LFQ intensity Cardioembolic**	**-log *P*-value**
SLC25A11	Q02978	Mitochondrial 2-oxoglutarate/malate carrier protein	Catalyzes the transport of 2-oxoglutarate at mitochondrial membrane	Mitochondrion	−1.278498	1.94
APOE	P02649	Apolipoprotein E	Lipid transport between organs via the plasma and interstitial fluids	Extracellular	−1.34310437	1.69
PHB	P35232	Prohibitin	Inhibits DNA synthesis	Mitochondrion	−1.1333107	2.46
ATP5A1	P25705	ATP synthase subunit alpha	Produces ATP from ADP	Mitochondrion, plasma membrane	−1.1749357	1.93

## Discussion

Our study advances the knowledge of the human cerebrovascular thombi composition by delivering the largest proteome dataset available to date. We focused on the protemic analysis of cardioembolic and atherothrombotic thrombi and we applied a support-vector machine learning routine in an exploratory, proof-of-concept, attempt to identify protein candidates segragating the two selected populations. Our research supports the general notion that direct analysis of the thrombi material could unveil, in the future, new disease players and candidate biomarkers potentially aiding stroke diagnosis. The SVM method used herein was set to identify combinations of protein trios (Table 2) in the intracranial thrombi, and it allowed for an 88.3% correct classification of our selected cardioembolic and atherothrombotic populations (Table 1). We here underscore that histological, cellular (e.g., red blood cells, platelets, white blood cells), and molecular (omics) analyses should be all integrated to obtain a complete and multi-level depiction of the thrombi structure and biology.

Understanding the composition of the human clot was previously attempted in two studies, although limited in sample size or lacking SVM analysis (12, 27) A first proteomic investigation correlated 2 inflammation-associated proteins (integrin alpha-M and mitochondrial superoxide dismutase) to high blood LDL (27). Mitochondrial superoxide dismutase was previously associated to unstable carotid plaques (28). These proteins were detected in our study, although without significant differences between cardioembolic and atherothrombotic thrombi. A second study analyzed 4 thrombi, with ~1,600 proteins identified (12). An earlier investigation, focused on human coronary thrombi in patients with ST-segment elevation in acute myocardial infarction, identified 708 proteins. The implication of platelet activation during the formation of thrombus causing acute coronary syndrome was suggested (29).

### Combining Mass-Spectrometry With SVM Analysis: Initial Feasibility and Proposed Applicability to Human Ischemic Stroke

An innovative aspect of the presented study is the methodological combination of large-scale proteomic tools and machine learning models or algorithms to define and potentially categorize the thrombi proteomes (3). In our patients' cohort, the fibrin stabilizing or coagulation Factor XIII (FXIII) was identified by SVM as one potential differentiating element between the cardioembolic and atherothrombotic thrombi analyzed (Table 2). FXIII is a key enzyme in the coagulation cascade that allows the cross-linking of fibrin chains with subsequent increase of mechanical clot strength and resistance to fibrinolysis (30). FXIII was also reported in embolized thrombi from the cardiac left atrial appendage in atrial fibrillation patients (31).

Interestingly, it has been recently shown that FXIII levels are higher after myocardial injury and that FXIII harbors an important role in cardiac healing and remodeling (32). Moreover, valine-to-leucine (V34L) single-nucleotide polymorphism (SNP), which is associated with higher levels of FXIIIa, appears to be associated with a lower risk of pathological thrombosis in ischemic heart disease (33, 34). Importantly, atrial fibrillation or atrial cardiopathies that share a common mechanism of thrombus formation in the left atrial appendage should be identified as soon as possible after stroke occurrence to initiate anticoagulation therapy (35). Our SVM learning analysis also identified proteins involved in the cellular cytoskeleton assembly (Table 2), namely the myosin light chain kinase and F-actin-capping protein. In general, the large scale proteomic analysis of human clots here executed discloses pathways and molecular players of clot-endothelium interplay and local inflammation related to cerebrovascular damage (Figure 2). The latter is important because cerebrovascular breakdown contributes to the development of central nervous system disease (6–8, 36), in this case potentially enabling post-stroke sequelae.

### Study Limitations and Prospectives

To further explore the utility of the protein candidates here discovered (Table 2) a validation step using an independent, and larger sample population will be necessary to define reproducibility and accuracy parameters (e.g., sensitivity, specificity, positive ad negative predictive values). Our SVM analysis, due to a relatively small sample size, only allowed accuracy estimation. A compelling question is whether our integrated proteomic-SVM method could be next used to examine specific signatures in case of cryptogenic stroke. We are aware that the proteins here identified may be not helpful in a population of cryptogenic stroke that includes etiologies other that the two studied here. We are aware that an efficient transition from SVM proteome analysis to clinical laboratory tools (e.g., Elisa) could be challenging and time consuming. (12, 27). The latter will be possible only when definitive molecular candidate(s) will be confirmed in larger populations with results replicated across stroke centers. Nevertheless, our study provide a proof of principle model that could be further developed and applied. Our proteome results (Supplementary Tables 1, 2) are shared and available to be re-analyzed using more advanced or alternative SVM methods.

We here recognize that the cohort used in the present study is heterogeneous in respect to age and blood platelet levels. Although blood platelet levels have been associated to stroke outcome (37), it is unknown whether a correlation with stroke etiology exists. One study showed that high platelet content of intracranial thrombi associates with large artery atherosclerosis. However, the authors did not study the correlation between blood platelet content and stroke cause (38). Another possible limitation of our approach concerns the retrieved material that may not represent the entire thrombus, although the analyses presented here were performed on the largest portion of clots retrieved at one pass of the thrombectomy device. IVT may also alter the samples, although this effect is likely to be limited due to the short time between IVT and thrombus extraction and processing. Finally, pre-stroke antithrombotic therapy may alter thrombus proteome composition (39).

### Conclusions

In summary, quantitative proteomics and SVM analysis can be feasibly combined to examine the variation of intracranial human thrombi proteomes. If further developed and tested on larger cohorts, these methods have the potential to discover precise and novel pathophysiological players and biomarkers, with the ideal goal of aiding cerebrovascular stroke diagnosis and secondary prevention.

## Data Availability Statement

The mass spectrometry proteomics data have been deposited to the ProteomeXchange Consortium via the PRIDE [1] partner repository with the dataset identifier PXD020398.

## Ethics Statement

The studies involving human participants were reviewed and approved by Comité de Protection des Personnes ≪Sud-Méditerranée IV≫, Centre Hospitalier Universitaire de Montpellier, hôpital Saint-Eloi, 34295 Montpellier Cedex 5. The patients/participants provided their written informed consent to participate in this study.

## Author Contributions

CD, JD, PM, VC, and NM: conception and design of the study, analysis of data, and drafting of the manuscript. EZ, MD, FB, and JL: acquisition and analysis of data, drafting of the manuscript, and figures. PH-L, GG, ID, CR, FC, and AB: acquisition of data. CD and VC: emergency surgery interventions, samples collection and patients’ approval. All authors contributed to the article and approved the submitted version.

## Conflict of Interest

The authors declare that the research was conducted in the absence of any commercial or financial relationships that could be construed as a potential conflict of interest.

## Supplementary Material

Supplementary Table 1 Complete list of proteins and mass-spectrometry data for each sample.

Supplementary Table 2 List of proteins commonly present in all samples.
